# Mobile Health App for Self-Learning on HIV Prevention Knowledge and Services Among a Young Indonesian Key Population: Cohort Study

**DOI:** 10.2196/17646

**Published:** 2020-09-08

**Authors:** Priyanka Rani Garg, Leena Uppal, Sunil Mehra, Devika Mehra

**Affiliations:** 1 MAMTA Health Institute for Mother and Child New Delhi India; 2 Medeon Science Park Malmo Sweden

**Keywords:** mHealth, Indonesia, HIV, key populations

## Abstract

**Background:**

Indonesia is the only country in the Asia Pacific region where the incidence of HIV is still on the rise, and its prevalence is extremely high among the key populations such as men who have sex with men, transgender women, and people who inject/use drugs. Mobile health (mHealth) apps provide an innovative platform for delivering tailored HIV prevention and care among these populations more efficiently than possible through the direct face-to-face approach.

**Objective:**

The aim of this study was to assess the role of a peer-customized mobile app based on the principle of self-learning for improving HIV prevention knowledge and access to health services among men who have sex with men, transgender women (known as Waria in Indonesia), and people who use drugs in Indonesia.

**Methods:**

A prospective intervention cohort study was conducted among the key populations in five provinces of Indonesia (Jakarata, West Java, East Java, Special Region of Yogyakarta, and Bali). The data were evaluated using a pre-post assessment survey conducted on a sample of 200 unique users, including 50 men who have sex with men and transgender women each, and 100 people who use drugs, with a follow-up response rate of 98% and 70%, respectively. An mHealth app named RUMAH SELA was developed and implemented among the key populations.

**Results:**

From baseline to the endpoint of the study, there was a significant increase in comprehensive HIV-related knowledge from 20% (10/49) to 60% (29/49), 22% (11/49) to 57% (28/49), and 49% (34/70) to 74% (52/70) among men who have sex with men (*P*=.004), transgender women (*P*<.001), and people who use drugs (*P*<.001), respectively. There was also a reduction in sexual activities without condom use from 22% (11/49) to 19% (9/49), 18% (9/49) to 12% (6/49), and 21% (15/70) to 10% (7/70) among men who have sex with men (*P*=.45), transgender women (*P*=.25), and people who use drugs (*P*<.001), respectively. There was an uptake of HIV testing by 31% (15/49) for men who have sex with men, 49% (24/49) for transgender women, and 26% (18/70) for people who use drugs after using the app. There was a reduction in injecting drugs with a used needle in drug users from 45/70 (78%) to 15/70 (26%). Measures of self-esteem increased among men who have sex with men (mean 26.4 vs mean 27.1), transgender women (mean 26.5 vs mean 27.8; *P*=.02), and people who use drugs (mean 24.0 vs mean 25.0). In addition, 27% (7/24) of men who have sex with men, 25% (4/15) of transgender women, and 11% (2/18) of drug users made an appointment for an HIV test through the app. The app was quite highly accepted by the key populations as nearly a quarter felt that they became more confident in discussing issues about sexuality, more than 80% found that the app provided sufficient knowledge about HIV, and more than half of the participants found the app to be user friendly.

**Conclusions:**

This one-of-a-kind mHealth intervention with an mHealth app as a self-learning tool is effective in increasing HIV-related knowledge and behavior, and access to services with strong acceptability by the community. There is a need to scale up such interventions for efficacy testing in a larger population to provide evidence for national-level mHealth programs addressing HIV.

## Introduction

Indonesia, situated in the southeast Asia region, is witnessing an upward trend in HIV/AIDS prevalence [1]. Indonesia is the only country in which the HIV epidemic is still on the rise among the cluster of five high-burden countries (India, Myanmar, Nepal, Thailand, and Indonesia) of the southeast Asia region [2]. The national HIV prevalence in Indonesia (among people aged 15 years and above) is 0.3% in the age group 15-49 years [3]. The HIV epidemic in Indonesia is mostly concentrated among key populations, including sex workers, men who have sex with men (MSM), and transgender women (known as *Waria* in Indonesia), although the prevalence varies across provinces [4]. The epidemic is on the rise particularly among MSM and their sexual partners. The HIV prevalence among MSM in Indonesia is 12.8% and is 7.4% for transgender women [5]. Among people who inject drugs, there has been a substantial decline in HIV prevalence from 52.4% in 2007 to 28.8% in 2015, although the absolute numbers are still quite high [6].

In 2012, the Indonesian government responded to the growing HIV epidemic with the continuum of care known as *Layanan Komprehensif Berkesinambungan,* which aimed at integrating prevention, care, and treatment for all, and directed individuals toward immediate antiretroviral therapy (ART) after receiving an HIV diagnosis; however, the coverage of prevention services among key populations remained less than 55% and only 10-20% of people living with HIV were receiving ART at the end of 2016 [5,6]. Current evidence indicates that less than half of the key populations with HIV in the Asia-Pacific region, including Indonesia, are aware of their HIV status and only around a third are referred for treatment [5]. Therefore, there is a pressing need to fill these gaps and move toward innovative and cost-effective solutions to address the health care needs for these populations.

The accessibility and potential of mobile health (mHealth) or electronic health are rapidly growing, and is considered a promising approach to reach young and key populations (including MSM, transgender women, and sex workers) for addressing health care delivery gaps in the Asian region [7-10]. Indonesia ranks among the top 10 countries in the mobile connectivity index. The 3G and 4G coverage has reached over 90% and 80%, respectively, with more than half of the country’s adult population now using mobile internet [11]. Encouragingly, there is evidence that online or mobile-based interventions are effective in cisgender young people, and are emerging for sexual minority youth as well; however, there are limited evidence-based interventions using mobile-based apps designed specifically for gender and sexual minority young people. Further, gamification has been viewed as a promising approach to enhance participant interaction, increase behavior change learning opportunities, and improve intervention appeal for adolescents and youth [12].

The aim of this study was to assess the role of a peer-customized mobile app based on the principle of self-learning in increasing knowledge on HIV transmission, creating awareness about safe sex and HIV prevention, facilitating behavioral change, and fostering the uptake of HIV testing for improving access to health services among the key populations, particularly among MSM, transgender women, and people who use drugs (PWUD), in Indonesia.

## Methods

### Participants

Participants were recruited from five provinces of Indonesia: Jakarta, West Java, East Java, Special Region of Yogyakarta, and Bali. Respondents were recruited with the help of peer leaders, who are community leaders/outreach workers associated with the program implementation organization. Peer leaders listed the key populations from their networks and mapped areas with hotspots. To qualify for the intervention, there were certain inclusion criteria defined for enrolling the participants: (1) aged between 16 and 30 years (identified by the Indonesian government as young adults) [13]; (2) identify themselves as MSM, transgender woman, or PWUD, including the use of cocaine, methamphetamine, or ecstasy in the last 12 months or in the last 4 weeks; (3) mobile phone literate and own an Android phone. A total of 200 unique users (50 MSM, 50 transgender women, and 100 PWUD) were identified to be eligible for the study and provided written informed consent (assent for those younger than 18 years). Consent was obtained from the guardians/caretaker/outreach worker for providing services to the minor participants. For each province, six peer leaders were identified who were responsible for mapping the app users, conducting the study, and monitoring the use of the app during the study period.

### Pre-Post Assessment

A baseline survey was carried out to assess the knowledge about HIV and sexual behavior of the participants in August 2017, followed by an endpoint survey conducted approximately 3 months into the intervention. Data collection was executed by the local Civil Society Organization based in Bandung with the help of peer leaders placed within the key population network (called Focus Muda) in Indonesia. A structured closed-ended questionnaire with special provisions to record open-ended responses wherever required was used in the data collection process. Consent and pre-post assessments were carried out in the local language (Bahasa). All procedures were approved by the Ethical Review Board at the Catholic University of Atmajaya in Jakarta, Indonesia.

### Intervention

#### Development and Design of the mHealth App

For the mobile app to resonate with the Indonesian key populations, in-depth interviews were conducted with a separate group of individuals who were members of the key populations (N=20). A comprehensive needs assessment to understand the knowledge and experience on HIV-related issues was conducted to determine the feasibility and acceptability of the app in the community. Each in-depth interview lasted for about 45 minutes and was conducted in Bahasa. All interviews were audio-recorded for subsequent transcribing. We conducted content analysis on the interview responses, and the data were coded to extract relevant themes related to the knowledge and experience on HIV-related issues of sexual health, safe sex, and risky behaviors among the key populations in Indonesia, and to identify ideal designing modalities for acceptability of the mobile app in the community. The themes assessed were the availability of smartphones among the respondents and their networks, to understand barriers in accessing services, and to determine the features of the app to inform intervention content, structure, design, and apt elements for a safe and secure mobile app.

#### Community-Supported Design Process

Following the formative assessment mentioned above, the app interface was designed. Six peer leaders among the 20 who were recruited from Focus Muda—a network of young people working on key population issues across Indonesia—designed the interface of the app in a workshop facilitated by MAMTA-Health Institute for Mother and Child, and the local implementing organization in Bandung. The peer leaders were identified according to preset criteria as community leaders/outreach workers. The main criteria were an association with the network partners, functional literacy and good communication skills, and experience of working with the targeted communities.

The mobile app design was created by peer leaders using a co-design approach [14]. A multistage design process was adopted to finalize the design and features of the app. Stage 1 involved initial assessment of the current situation related to the versions of Android phones under use by the study participants, and to screen resolutions, network connectivity issues, one-time password gateway integration, and related issues. Stage 2 involved having informal discussions with peers on the type of mobile apps regularly used, approximate time spent online, and top-rated features for providing a mobile experience. Stage 3 involved designing the user interface and online avatars for the various features proposed by the peers. Sketching and prototyping the app was then started, and the beta version of the app was tested by different constituencies, including the peers, project implementation team, and technical partners.

#### Integrated Team of Health Care Providers

A medical doctor with experience in sexually transmitted infections, HIV/AIDS treatment, and management was empaneled with the app. A young primary health care worker with extensive experience in providing HIV counseling and testing services was included within the referral options of the app. Prior approval and consent from the health care providers were obtained before they became involved in the project. The health care providers were given access to the app dashboard (ie, a protected site with a user ID and password) to view the questions asked by the users and respond appropriately within 12 hours. The users were not prescribed medicines online through this app, and the responses were restricted to addressing clarifications and doubts about any of their concerns, including signs and symptoms of HIV/AIDS and other sexually transmitted diseases such as hepatitis C, drug use and rehabilitation, and masturbation.

#### App Content

The content included information on the transmission, prevention, and treatment of sexually transmitted infections with a special focus on HIV/AIDS, myths and misconceptions regarding HIV/AIDS, correct condom use, the importance of HIV testing, and access and early linkage to HIV testing services. The app content was focused on these themes as evidence indicates the need to intervene early for increased awareness generation among these populations [15]. The content was provided in two languages, Bahasa and English, and was linked to the internet.

#### Technical Platform

**Figure 1:**
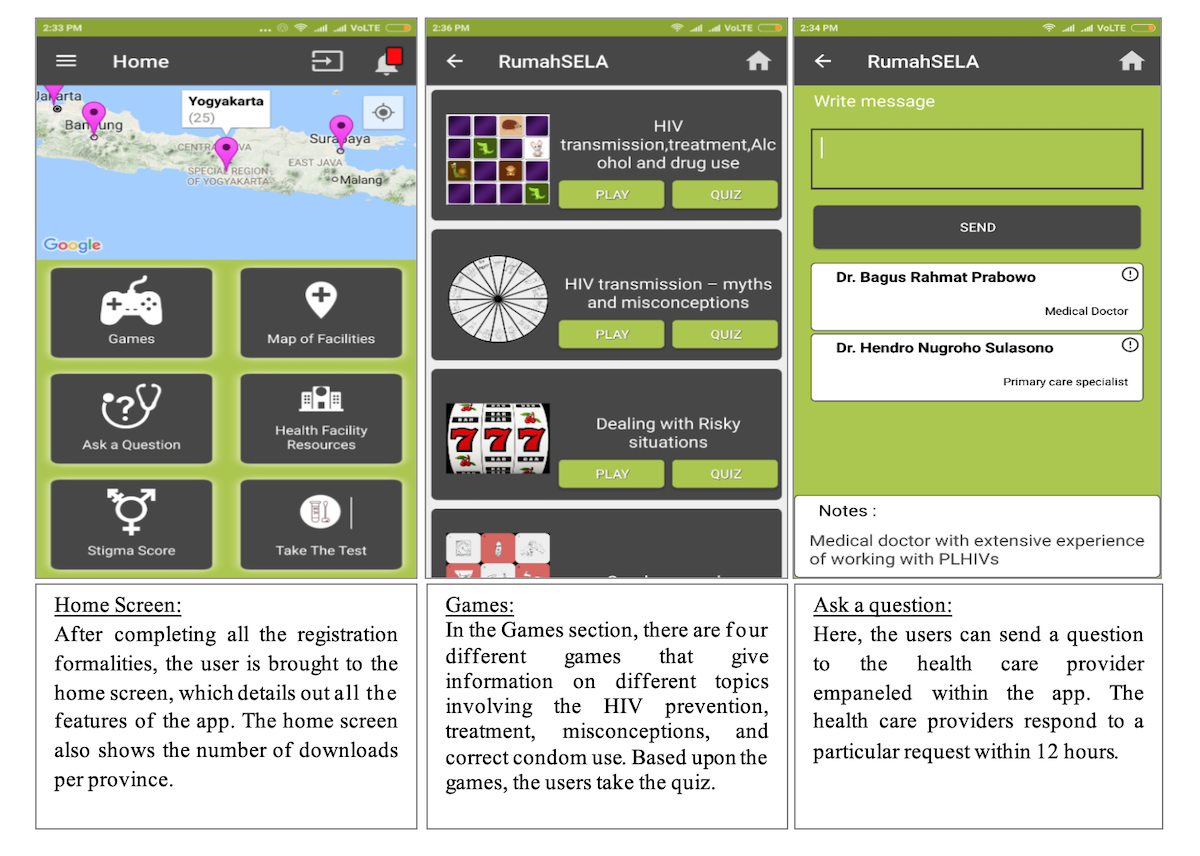
Interface of key features of the app.

The final Android-based mobile app was developed by a software development company based in India. The field test of the app was conducted for removal of any bugs and irrelevant content/features before distribution. The mobile app was distributed to each individual’s phone through a link, which was secured from the software development company. Registration of the app users, consent, password setting, risk assessment, home screen, ask a question, map of health facilities, request for test intimation, instant in-app “ask a question” facility, stigma information, feedback on the app experience, condom games, quiz, and prize were the key features built into the app. Some screenshots showing the interface of the key features of the app are shown in Figure 1.

The ask-a-question feature helped the users to send private messages to the health care providers empaneled with the app. The health care providers responded to the particular request within 12 hours. Based on the need of every participant, the link to health services was provided to each user through the built-in map of health facilities and the list of health facility resources. This helped the users to easily connect with the health facilities, particularly aimed at improving the linkage and effective utilization of health services. The administration of the app was peer-facilitated, and the participants were reached with a staggered approach. Focus Muda nominated 23 youth peer leaders to administer the mobile app.

### Measures

#### Demographics

Demographic variables included age, gender, sexual orientation, relationship status, level of education, current employment, income, living situation, and drug abuse behavior.

#### HIV/AIDS Knowledge

We assessed the comprehensive knowledge on HIV, adopted and modified from Ankunda and Asiimwe [16], which refers to the knowledge of reducing HIV risk by consistent use of condoms and being faithful to one uninfected sexual partner who has no other partners, and included a rejection of misconceptions of HIV transmission through a mosquito bite and sharing a meal with an HIV-infected person [16]. We considered the knowledge on the first two indicators, and then included the rejection of misconceptions of sharing utensils and clothes with an HIV-infected person and social kissing an HIV-infected person as comprehensive knowledge. This was assessed from baseline findings, which highlighted these myths and misconceptions to be prevalent in the studied key populations. Furthermore, Ankunda and Asiimwe [16] used the knowledge on HIV transmission from an HIV-infected mother to a child as another set of assessment of comprehensive knowledge. During our analysis, we found that knowledge on this indicator was poor in the studied key populations. Therefore, this was adopted as the fifth indicator of comprehensive knowledge on HIV in the current study.

#### Sexual Behavior

To assess HIV risk behaviors, we asked participants about the number of times they had anal, vaginal, insertive, and receptive sexual intercourse, and whether they had sex with and without a condom with different partners (casual sex with a male or female, transgender male or female) during the last 3 months.

#### Recency of HIV Testing

We asked the participants “When did you last have an HIV test?” In addition, to assess the effect of the mHealth app on HIV testing, we asked the participants “Did you go for an HIV test after using the app?”

#### Self-Esteem

We adopted the 10-item Rosenberg Self-Esteem Scale to measure the self-esteem of the participants during the study [17]. The answers to negative items such as “At times, I think I am no good at all” were reverse scored. The scores ranged from 0 to 30 with higher scores indicating higher self-esteem. Cronbach α was .81 and .72 during the pre-post assessments, indicating strong reliability.

#### Mobile App Acceptability

We also assessed (1) the feasibility of using the mHealth app to receive information on sexual health and HIV (eg, “On what aspects did you receive knowledge through the app?”); (2) perceived impact of using the mHealth app (eg, “Has the app made any impact on your knowledge or behavior? If yes, how has the app made an impact?”); (3) quality of the medico-client relationship (eg, “Did you send a chat message through the app to get health information? If yes, what kind of health and other information did you seek? Did you feel the response was satisfactory?” [with response options always, sometimes, never] “Did you send a request for booking an appointment for an HIV test with the doctor through the app?”); and (4) general perceptions about the features of the app (eg, “Which feature of the app was most useful in having an impact?” “How would you rate the ease of use of the app?”).

#### Pre-Post Assessment

Descriptive statistics were computed for demographic characteristics. We applied the McNemar test to assess the possible shift from the baseline to the endpoint for all outcomes. The shift is presented as a percentage point change. Statistical significance was considered at *P*≤.05. All analyses were carried out using SPSS 22 (IBM Corp, Armonk, NY, USA).

## Results

### Participation and Intervention Completion Rates

A total of 200 unique users (50 MSM, 50 transgender women, and 100 PWUD) of the app were identified based on the eligibility criteria. Thirty-two participants from the MSM and transgender groups, and 30 from the PWUD group were lost to follow up. Therefore, the follow-up response rate was 98% in MSM and transgender women and was 70% in the PWUD. More than 90% of the dropouts among the PWUD group were injecting drug users.

### Sociodemographic Profile

Table 1 summarizes the sociodemographic characteristics of the participants. The mean age was in the low to mid-20s for the three groups. The majority of the PWUD were males. The majority of the participants reported being single at the time of the study. More than half of the participants were educated up to a high school level. More than half of the MSM were working in private sectors, whereas about half of the transgender women were engaged in sex work, followed by singing and dancing. Nearly one-quarter of the PWUD were involved in sex work and over a quarter of the PWUD were working in the private sector. Nearly half of the MSM and about three-quarters of the PWUD reported living with family, whereas the majority of the transgender women lived alone.

The majority of the PWUD reported ever injecting drugs, with the majority indicating that they had injected drugs in the last month and have a history of sharing a needle.

**Table 1:** Sociodemographic characteristics of the participants (N=168).

Characteristics		Men who have sex with men (n=49)	Transgender women (n=49)	People who use drugs (n=70)
Age (years), mean (SD)		22.6 (3.3)	25.6 (3.0)	23.7 (4.0)
**Sex, n (%)**				
	Male	49 (100)	0	56 (80)
	Female	0 (0)	0	14 (20)
	Transgender	0 (0)	49 (100)	0
**Marital status, n (%)**				
	Single	49 (100)	45 (92)	56 (80)
	Partnered	0 (0)	4 (8)	14 (20)
**Educational level, n (%)**				
	No education	0 (0)	1 (2)	0 (0)
	Elementary (grades 1-6)	0 (0)	1 (2)	5 (7)
	Middle school (grades 7-9)	1 (2)	14 (29)	13 (19)
	High school (grades 10-12)	34 (69)	31 (63)	47 (67)
	Technical college/university	14 (29)	2 (4)	5 (7)
**Employment, n (%)**				
	Private job	34 (68)	9 (18)	18 (26)
	Government job	2 (4)	0 (0)	0 (0)
	Self-employed	3 (6)	6 (12)	13(19)
	Singing/Dancing	0 (0)	12 (24)	2 (3)
	Sex work	1 (2)	26 (52)	20 (29)
	Unemployed	10 (20)	7 (14)	9 (1)
**Living situation, n (%)**				
	Living with parents/family	25 (51)	7 (14)	52 (74)
	Living with male partner	6 (12)	2 (4)	5 (7)
	Living alone	17 (35)	39 (80)	9 (13)
	Living with friends/others	1 (2)	1 (2)	4 (6)
**Drug use, n (%)**				
	Ever taken drugs	1 (2)	5 (10)	70 (100)
	Ever injected drugs	0 (0)	2 (4)	58 (83)
	Injected drugs in prior 1 month	0 (0)	1 (2)	48 (83)^a^
	History of needle exchange	0 (0)	0 (0)	41 (71)^a^

^a^Proportion calculated among those who ever injected drugs.

### Correct Knowledge on HIV

#### Modes of HIV Transmission

Table 2 shows the shift in correct knowledge about the modes of transmission of HIV/AIDS from baseline to the endpoint of the study among the three key populations. The majority of the participants were aware of sex with multiple partners, unprotected sex with an HIV-infected person, blood transfusion from an HIV-infected person, and sharing an HIV-infected needle/syringe as the modes of transmission at baseline. Use of the mobile app resulted in a significant increase in the knowledge about HIV transmission from a mother to child with a percentage point change of 16.4%, 36.7%, and 27.1% among the MSM, transgender women, and PWUD, respectively, from baseline to the endpoint.

**Table 2:** Shift in correct knowledge about HIV/AIDS from baseline to the study endpoint among the key populations at risk for HIV in Indonesia.

Characteristic		Men who have sex with men (n=49)				Transgender women (n=49)					People who use drugs (n=70)			
		Baseline, n (%)	Endpoint, n (%)	PP^a^ change	*P* value	Baseline, n (%)	Endpoint, n (%)	PP change	*P* value	Baseline, n (%)		Endpoint, n (%)	PP change	*P* value
**Modes of transmission**														
	Having sex with multiple partners	47 (96)	49 (100)	4	.16	45 (92)	49 (100)	2	.56	66 (94)		67 (96)	1	>.99
	Having unprotected sex with an HIV-infected person	49 (100)	49 (100)	0	.32	49 (100)	49 (100)	0	.08	69 (99)		70 (100)	1	>.99
	Having blood transfusion from an HIV-infected person	49 (100)	49 (100)	0	>.99	46 (96)	49 (100)	4	.50	70 (100)		70 (100)	0	.50
	Sharing HIV-infected syringes or needles	49 (100)	49 (100)	0	>.99	42 (86)	46 (94)	8	.22	68 (97)		70 (100)	3	.50
	From HIV-infected mother to her baby	40 (82)	48 (98)	16	.004	26 (53)	44 (90)	37	<.001	38 (54)		57 (81)	27	.003
**Myths and misconceptions about HIV transmission**														
	HIV can be contracted through mosquito bites	46 (94)	45 (92)	–2	>.99	43 (88)	40 (82)	–6	.51	63 (90)		67 (96)	6	.13
	HIV can be contracted by sharing utensils and clothes with an HIV-infected person	44 (90)	45 (92)	2	>.99	45 (92)	49 (100)	8	.13	56 (80.0)		66 (94)	14	.01
	HIV can be contracted by living and sharing a meal with an HIV-infected person	36 (92)	47 (96)	4	.63	27 (90)	48 (98)	8	.50	67 (96)		68 (97)	1	>.99
	HIV can be contracted by kissing someone who is infected	39 (80)	42 (86)	6	.45	37 (76)	45 (92)	16	.008	56 (80)		63 (90)	10	.04
	HIV can be contracted by using public toilets/bathrooms	46 (94)	47 (96)	2	>.99	46 (94)	47 (96)	2	>.99	63 (90)		68 (97)	7	.13
**Prevention of HIV**														
	Using condoms during sexual contact	45 (92)	45 (92)	0	>.99	44 (90)	47 (96)	6	.45	55 (79)		67 (96)	17	.004
	Having sex with only one faithful and uninfected partner	41 (84)	89.8	6	.38	28 (57)	38 (78)	21	.02	46 (66)		55 (79)	13	.03
	Taking injections using clean and unused syringes	36 (74)	37 (76)	2	>.99	23 (47)	29 (59)	12	.21	55 (79)		63 (90)	11	.02
**HIV treatment**														
	Heard of ART^b^ as a treatment of HIV	14 (40)	31 (78)	38	<.001	11 (27)	37 (84)	57	.002	34 (51)		50 (75)	24	<.001
	ART can help reduce viral load	30 (61)	35 (71)	10	.56	34 (69)	35 (71)	2	.32	35 (50)		45 (64)	14	.27
	ART can help improve quality of life	22 (63)	29 (73)	10	.32	29 (59)	31 (74)	15	.66	22 (31)		41 (9)	27	<.001

^a^PP: percentage point.

^b^ART: antiretroviral therapy.

#### Myths and Misconceptions

The increase in the awareness that HIV does not spread by sharing utensils and clothes was most significant among PWUD with a percentage point change of 14%. Over three-quarters of transgender women and PWUD reported that a person can contract HIV by kissing someone who is infected before the intervention. There was a significant change in the level of awareness with regard to this myth postintervention, with a percentage point change of 16% and 10% for transgender women and PWUD respectively reporting the correct information (Table 2).

#### HIV Prevention

Over three-quarters of the PWUD correctly reported that condom use can prevent HIV transmission before the intervention, and this knowledge further increased after the intervention with a percentage point change of 17% (Table 2).

#### HIV Treatment

Knowledge on ART as the basic line of treatment for HIV significantly increased among all groups postintervention by 38%, 57%, and 24% among MSM, transgender women, and PWUD, respectively (Table 2).

#### Comprehensive HIV Knowledge

There were significant shifts in comprehensive knowledge on HIV from 20% (10/49) to 60% (29/49), 22% (11/49) to 57% (28/49), 49% (34/70) to 74% (52/70) among MSM (percentage point change of 40%, *P*=.004), transgender women (percentage point change of 35%, *P*<.001), and PWUD (percentage point change of 25%, *P*<.001), respectively, representing a total change from 29% to 63% for all key populations.

#### HIV-Related Risk Behaviors

There was a reduction in the number of individuals who did not use a condom in their last sexual intercourse encounter postintervention in all three groups (with a maximum shift in the PWUD). Among PWUD who abused drugs in the form of injection, the number of individuals who reported injecting drugs with a used needle or syringe in the last month reduced by over 50% after the intervention (Table 3).

**Table 3:** High-risk behaviors among key populations in the baseline and endpoint surveys.

Indicators	Men having sex with men (n=49)				Transgender women (n=49)					People who use drugs (n=70)			
	Baseline, n (%)	Endpoint, n (%)	PP^a^ change	*P* value	Baseline, n (%)	Endpoint, n (%)	PP change	*P* value	Baseline, n (%)		Endpoint, n (%)	PP change	*P* value
												
Not using condoms at the last sexual intercourse	11 (22)	9 (19)	–3	.45	9 (18)	6 (12)	–6	.25	15 (21)		7 (10)	–11	<.001
Injecting with a used needle or syringe in the past 3 months	0	0	0	N/A^b^	1 (2)^c^	0	–2	N/A	45 (78)		15 (26)	–52	<.001

^a^PP: point percentage.

^b^N/A: not applicable.

^c^N=1 among transgender women who abused drugs in the form of injection; therefore, no statistics were computed.

### Access to HIV Testing Services

There was a significant increase in the uptake of HIV testing after using the mHealth app from 79% (133/168) to 90% (151/168) before and after the intervention with a percentage point change of 11% (*P*<.035) for the three groups combined. The shift was the highest in the PWUD group, from 65% (46/70) before the intervention to 82% (57/70) after the intervention with a percentage point change of 17% (*P*<.001), followed by MSM (from 88% to 94%) and transgender women (96% to 98%).

Overall, 34% of the study participants reported that they went for an HIV test after using the app, 21% (35/168) of whom had made the appointment through the app. Almost half (24/49, 49%) of the transgender women, and 31% (15/49) and 26% (18/70) of the MSM and PWUD reported that they went for an HIV test after using the app. Of those who reported going for an HIV test after using the app, 27% (7/24) MSM, 25% (4/15) transgender women, and 11% (2/18) PWUD had made the appointment through the app.

### Self-Esteem

There was a positive shift in self-esteem from baseline to the endpoint in all groups with a significant shift among the transgender women (Table 4).

**Table 4:** Self-esteem scores among the key populations in the baseline and the endpoint surveys.

Population	Baseline, mean (SD)	Endpoint, mean (SD)	*t* value	*P* value
Men who have sex with men (n=49)	26.44 (3.66)	27.06 (3.41)	0.873	.39
Transgender women (n=49)	26.54 (2.83)	27.84 (2.38)	–2.468	.02
People who use drugs (n=70)	24.04 (5.45)	25.04 (3.58)	–1.324	.19

### Acceptability of the App

The majority of the participants (36/39, 74% MSM; 41/49, 84% transgender women; and 47/70, 67% PWUD) stated that they liked the game and health facility map features of the app. Over one-quarter (13/49, 26%) MSM and transgender women each, and 16% (11/70) of the PWUD asked a question through the app to get health information. The most common type of information sought was knowledge about HIV. The majority of the participants (40/49, 82% MSM; 41/49, 84% transgender women; 59/70, 84% PWUD) felt that the app provided knowledge on HIV with respect to risk, safer sexual practices, and prevention and treatment of HIV. Nearly a quarter of the participants (11/49, 22% MSM; 10/49, 20% transgender women; 16/70, 23% PWUD) said that they became more confident in discussing issues on sexuality after using the app. Nearly half of the participants (28/49, 57% MSM; 15/49, 31% transgender women; 41/70, 59% PWUD) found the app neither difficult nor easy to use, while 33% (16/49) MSM and transgender women each, and 20% (14/70) PWUD found the app easy to use. Further, only 23% (39/168) of the total participants had used any other mobile app before RUMAH SELA.

## Discussion

### Principal Findings

This study tested the potential use of an mHealth app (RUMAH SELA) as a self-learning tool for HIV prevention, which was developed and designed internally, and customized for key populations (MSM, transgender women, and PWUD) in Indonesia with the support of peer leaders. This intervention shows potential promise for enhancing the spectrum of HIV knowledge, reduction in risky sexual behaviors, uptake of services, and improving self-esteem among these vulnerable and underserved groups.

The comparison of outcomes before and after the intervention showed marked improvement in HIV-related knowledge, testing, and behavioral outcomes (eg, condom use and not injecting drugs using syringes/shared needles among PWUD). There was a potential uptake of services after the intervention. This study showed that 34% of the participants took an HIV test after using the app. A significant reduction was observed in the number of individuals who did not use condoms in their last sexual intercourse encounter postintervention in all three groups. PWUD showed a marked reduction in injecting drugs using a shared needle after using the mHealth app. Similar intervention strategies such as internet-based, text messaging, and smartphone apps were also found to be successful in reducing substance abuse in studies conducted in upper-middle and high-income countries such as Romania, the United States, Germany, and Switzerland in 2017 [18,19].

We found that certain myths regarding the transmission of HIV through a mosquito bite, social kissing, and sharing common utensils/clothes/bathroom/meal with an infected person were prevalent among the key populations, with the spread of HIV through social kissing as the most prevalent myth believed. This mHealth intervention could successfully dispel these myths.

There was a 3%, 6%, and 11% reduction among MSM, transgender women, and PWUD, respectively, who did not use a condom in their last intercourse after the intervention, indicating the potential value of the mHealth app in enhancing safer sexual practices. The study findings are in line with other studies that have discerned the impact of mHealth interventions in improving the sexual and behavioral health of vulnerable and underserved populations [12,18,20,21].

Although assessment of mental health was not within the scope of the present intervention, we tried to assess the effect of the app on self-esteem of the participants. It is noteworthy that the use of the mHealth app in this intervention could help to raise the self-esteem of the participants. Self-esteem is a precursor to both physical and mental health. Further, it acts as a protective factor contributing to positive social behavior [22]. Improving the self-esteem of an underserved population may have a benefit by acting as a catalytic agent for behavior change and consequently contributing to increased seeking for HIV services, prevention, and care.

In addition to behavioral interventions, the combination of access to HIV services along with behavioral change is an important strategy for HIV prevention and care. Most existing mobile-based interventions are contextualized particularly to improve the medical adherence to ART for people living with HIV [13,23-25]. Few qualitative studies have emphasized the use of mHealth for promoting the uptake of HIV testing [16,26]. This study highlights the utility of an mHealth app designed on the principles of community engagement in not only creating awareness about safer sex and increasing knowledge about HIV prevention, transmission, and treatment, but also in improving the uptake of HIV services among young key populations. A change was also found in terms of users having learned about the location of health service providers and the self-assessment of risk for HIV. The features of the app that were deemed to have the most positive impact were the map of health service facilities, games, and the ability to share your story, aside from acknowledging the user-friendliness of the app as key aspects that highlighted app acceptability by the target population.

### Strengths and Limitations

One of the strengths of this study is that it is a peer-customized mHealth app useful for HIV interventions based on a self-learning principle with minimal manual involvement. However, the findings of this study need to be considered in the context of certain limitations. The small sample size was one of the major limitations of this study; we assume that a larger sample could have provided more statistically meaningful inferences. The retention rate was 98% among MSM and transgender women but was only 70% among PWUD.

This study did not include any control group, which limits the validation of the intervention’s efficacy in changing the sexual and behavioral health outcomes. Further, based on the formative results, Facebook, Twitter, Instagram, Lollipop, Grinder, and Hornet were listed as apps/social media platforms that the participants had already used. These apps could be other possible potential sources of information for these populations apart from the RUMAH SELA app. However, the longitudinal nature of the study did provide an opportunity to test the efficacy of the intervention. Another limitation of the study was that it was a short-term intervention with an endpoint assessment conducted after 3 months. An intervention conducted for a longer duration with periodic assessments may have yielded more informative results. Since the app was designed and customized for the key populations of Indonesia, its utility may not be generalized for a general population. Lastly, we could not assess the impact of the sociodemographic parameters on the intervention for the differential outcomes related to the utility of the app across the groups due to the small sample size and thus insufficient statistical power for inference. Nevertheless, the app was found to be fairly well accepted by users. Moreover, only less than a quarter of the study participants had used any other mobile app before RUMAH SELA, which supports to a certain degree that the shift in knowledge and health-seeking behaviors among the study participants was directly due to exposure to RUMAH SELA.

### Conclusions

The RUMAH SELA mHealth app opens a window for enhancing knowledge on HIV and related behavioral outcomes among key populations with minimal external support and involvement, thereby offering a cost-effective and self-administered intervention package. This calls for larger intervention studies/trials, small-scale efforts, and pilot studies that must begin to scale with a rigorous design for future evaluation. These efforts will strengthen and provide evidence for program leaders, policymakers, and funders to develop influential population-based mHealth intervention strategies to combat HIV.

## Abbreviations

ART: antiretroviral therapy
mHealth: mobile health
MSM: men who have sex with men
PWUD: people who use drugs
